# Pathological Role of Tonsillar B Cells in IgA Nephropathy

**DOI:** 10.1155/2011/639074

**Published:** 2011-07-18

**Authors:** Yusuke Suzuki, Hitoshi Suzuki, Junichiro Nakata, Daisuke Sato, Tadahiro Kajiyama, Tomonari Watanabe, Yasuhiko Tomino

**Affiliations:** Division of Nephrology, Department of Internal Medicine, Juntendo University Faculty of Medicine, Hongo 2-1-1, Bunkyo-ku, Tokyo 113-8421, Japan

## Abstract

Although impaired immune regulation along the mucosa-bone marrow axis has been postulated to play an important role, the pathogenesis of IgA nephropathy (IgAN) is unknown; thus, no disease-specific therapy for this disease exists. The therapeutic efficacy of tonsillectomy or tonsillectomy in combination with steroid pulse therapy for IgAN has been discussed. Although randomized control trials for these therapies are ongoing in Japan, the scientific rationale for these therapies remains obscure. It is now widely accepted that abnormally glycosylated IgA1 and its related immune complex (IC) are probably key molecules for the pathogenesis, and are thus considered possible noninvasive biomarkers for this disease. Emerging evidence indicates that B cells in mucosal infections, particularly in tonsillitis, may produce the nephritogenic IgA. In this paper, we briefly summarize characteristics of the nephritogenic IgA/IgA IC, responsible B cells, and underlying mechanisms. This clinical and experimental information may provide important clues for a therapeutic rationale.

## 1. Introduction

IgA nephropathy (IgAN) is the most common form of glomerulonephritis (GN) globally, accounting for 25%–50% of primary GN patients [1]. Long-term follow-up studies have shown that up to 25%–30% of IgAN patients progress to end-stage kidney disease within 20–25 years [2]. However, the pathogenesis of IgAN remains unclear, and consequently, no disease-specific therapy for IgAN exists.

The recurrence of IgA deposition in renal allografts [3] and the disappearance of IgA deposits from renal allografts taken from donors with undiagnosed IgAN [4, 5] reinforce the importance of systemic abnormalities of the IgA immune system in IgAN, arguing against IgAN being a disease limited to intrinsic renal abnormalities. Several clinical studies have identified the importance of IgA or IgA-IC deposition as a fundamental causative factor in IgAN [6]. The observed clinicopathological heterogeneity may, at least in part, be dependent on the characteristics of the deposited IgA-IC itself or changes in the IgA immune system, including sites of IgA synthesis and stimulation and regulation of immunecompetent cells involved in the production of IgA [7].

On the other hand, the episodic macrohematuria, coinciding with mucosal infection such as tonsillitis and pharyngitis [8] or an abnormal response to mucosal vaccination in IgAN patients [9, 10], indicates that dysregulation of the mucosal immune system may play an important role in the pathogenesis of IgAN [6]. In addition, tonsillectomy is effective in long-term renal survival in IgAN patients [11]. Some Japanese studies have recently reported that tonsillectomy in combination with steroid pulse therapy can be a more effective therapy for IgAN than tonsillectomy alone [12–14]. 

However, the therapeutic validity of tonsillectomy and the indication for tonsillectomy for IgAN are controversial [15–17], even in Japan. Although tonsillectomy in certain patients can be an effective therapy, 7%–10% of IgAN patients show spontaneous clinical remission [18]. Therefore, a rationale and reasonable clinical markers are needed for indication of this therapy. Recent studies show that predictive factors for resistance to tonsillectomy in combination with steroid pulse therapy are age of onset, severity of proteinuria and hematuria, and pathological grade [19]. Although there is an ongoing randomized control trial evaluating the effect of tonsillectomy on this disease, mainly by the Special Study Group on Progressive Glomerular Disease of the Ministry of Health, Labor, and Welfare of Japan and the Japanese Society of Nephrology, the results are not yet available.

Many reports demonstrate that tonsillectomy is an effective therapy for dermatological diseases such as pustulosis palmaris et plantaris and psoriasis, sternocostoclavicular hyperostosis, and rheumatoid arthritis [20–23]; the rationale for this effect is also unknown. In contrast, elucidation of the rationale in IgAN may provide conclusive clues for the pathogenesis of not only IgAN but also the so-called “tonsillar focal infectious diseases.” To assess that rationale, we briefly summarize the characteristics of nephritogenic IgA and the B cells responsible for producing the nephritogenic IgA.

## 2. Generation of Nephritogenic IgA in the Mucosa-Bone Marrow (BM) Axis in IgAN

High levels of higher molecular forms of IgA are present in the serum of IgAN patients [2, 6, 7]. In addition, it is generally accepted that IgA deposits in glomerular mesangium primarily consist of polymeric forms of IgA1 including IC [2, 6, 7, 24]. Large numbers of polymeric IgA- (pIgA-) positive plasma cells are found in BM in IgAN [6, 7, 25]. Moreover, BM transplantation (BMT) in leukemia and IgAN patients has resulted in curing not only of leukemia but also of IgAN [26, 27], suggesting that overproduction of nephritogenic pIgA1 in IgAN seems to be partly based in systemic immune sites, such as BM. Furthermore, mucosal vaccination results in impaired mucosal IgA responses in IgAN whereas systemic antigen challenge results in increased titers of circulating pIgA1 antibodies with normal levels in mucosal secretions [28, 29]. In addition, not only IgA^+^ cells but also polymeric IgA are increasingly produced in the mucosa of IgAN patients [30–32]. On the other hand, there is a report demonstrating that there is a reduction in polymeric IgA producing cells in duodenum [33]. Therefore, the crosstalk between the mucosa and BM should be carefully discussed. Furthermore, we should remember that the tonsil has distinctive immunophenotypic characteristics and immune status including anatomical location, and population, and/or distribution of lymphocytes from the rest of the mucosal immune system. 

In a series of studies published almost 20 years ago, Van Es et al. identified impaired IgA immune responses in the mucosa-BM axis in IgAN [25, 29, 34, 35]. In the last decade, clinical and experimental studies have revealed continued trafficking of antigen-specific lymphocytes and antigen-presenting cells between the mucosa and BM in humans [6, 36]. The patterns of integrins and chemokine receptors in these lymphocytes, including memory B cells and IgA plasma cells, are slightly different and depend on the site of induction. This migration is directed by the local synthesis of specific chemokines and appropriate adhesion/homing-receptor engagement. There is increasing recognition for the presence of a mucosa-BM axis in humans, and abnormalities in this estimated axis may play an important role in the development of IgAN [6].

## 3. Characteristics of Abnormally Glycosylated IgA in IgAN

It is now apparent that serum levels of galactose-deficient IgA1 (GdIgA1), mainly in IC forms, are often elevated in IgAN patients [37–39]. Moldoveanu et al. recently demonstrated, using a lectin-binding assay, that Caucasian IgAN patients have increased levels of serum GdIgA1 [37]. The mesangial IgA deposits also display abnormal *O*-glycosylation [40, 41] in IgAN. It is known that underglycosylated IgA tends to be aggregated, indicating that the polymeric formation of IgA1 may be, at least in part, based on the aberrant glycosylation of IgA1 [42–44]. Furthermore, pIgA has more capacity to activate mesangial cells than monomeric IgA [45–49]. Accordingly, the involvement of GdIgA in the pathogenesis of IgAN has been discussed [2, 50, 51]. A recent study has shown that IgA1 secreted by Epstein-Barr virus-immortalized B cells from peripheral blood of IgAN patients was mostly polymeric, and had galactose-deficient sialylated *O*-glycans due to decreased *β*1, 3-galactosyltransferase (core1*β*GalT) activity, and increased *N*-acetylgalactosamine-specific *α*2,6-sialyltransferase activity [38]. On the other hand, IgA1 produced by tonsillar lymphocytes is also abnormally glycosylated in IgAN patients [52–55]. These clinical findings taken together further support the idea that the estimated mucosa–BM axis is the location where B cells producing the nephritogenic GdIgA1 may traffic between tonsil and other organs via peripheral blood in human IgAN [36].

Humans have two isotypes of IgA: IgA1 and IgA2. IgA1 contains *O*-glycosylation sites but IgA2 or murine IgA do not [56]. Thus, the murine model does not include the aberrant *O*-glycosylation involved in the human IgAN. However, recent studies suggest that aberrant glycosylation of *N*-glycans may be involved in the pathogenesis of murine IgAN [57, 58], suggesting that aberrant modifications of carbohydrates of serum IgA are involved in the development of not only human but also murine IgAN, whether the carbohydrates are *O*-glycans or *N*-glycans. 

Renal biopsy and subsequent immunohistochemical analysis of the renal tissue remains the gold standard for diagnosing IgAN or evaluating the activity of acute lesions of this disease; however, new sensitive, and reasonably specific, non-invasive tests are emerging and may provide another diagnostic approach. Our recent study showed that reduction of serum GdIgA1 after tonsillectomy was associated with improvement of hematuria in certain IgAN patients (our unpublished data). Since the primary abnormal clinical manifestation in IgAN is recurring bouts of hematuria with or without associated proteinuria [59], elevated circulating levels of GdIgA1 is one of the most promising new tests for diagnosis or activity of IgAN [37, 40, 41, 52–54]. 

However, Gharavi et al. have recently reported that GdIgA1 levels were increased in 78% of sporadic IgAN patients and in 25% of their blood relatives, although the majority of relatives with abnormally glycosylated IgA1 were asymptomatic [60]. This finding suggests that, in certain cases, additional cofactors are required for development of IgAN. In a recent publication, Suzuki and coworkers described the characteristics of IgG autoantibodies to abnormally glycosylated IgA1 secreted by immobilized B cells derived from sporadic IgAN patients [61]. The serum levels of these IgG autoantibodies correlated closely with the degree of proteinuria, suggesting that IC formation of aberrantly glycosylated IgA and glycan-specific IgG antibodies and subsequent enlargement of their molecular mass may be an additional cofactor required for full development of the disease [62, 63]. In this regard, it is noteworthy that the serum levels of IgA-IgG2a IC, but not of IgA, were shown to correlate closely with the severity of glomerular lesions in IgAN-prone mice [64]. In addition to the aberrant glycosylation of IgA, similar mechanisms may underlie the progression of both human and murine IgAN.

## 4. Mucosal Encounter of Nephritogenic Exogenous Antigens in IgAN

The association of episodic macroscopic hematuria with mucosal infections in IgAN is suggestive of changes to the mucosal immune system in this disease, which may include changes in antigen handling [6]. The results of immunization studies in IgAN patients support that notion. Mucosal immunization with neoantigen results in impaired mucosal and systemic IgA responses but normal IgG and IgM responses [9, 10], suggesting that in IgAN there is mucosal hyporesponsiveness to mucosal neoantigens. In contrast, systemic and mucosal immunization with recall antigens results in exaggerated systemic IgA responses with increased and prolonged production of specific IgA [11, 12, 28, 65]. These results suggest that IgAN patients respond excessively to recall antigens [6].

Common microbial and food or food-borne antigens may also play a role in this process. Emancipator et al. [66] demonstrated a pathogenic relationship between prolonged mucosal antigenic exposure, formation of circulating IgA-IC, and the development of GN. Those authors orally immunized Balb/c mice with protein antigens and found a significant increase in specific IgA-producing plasma cells in the lamina propria of bronchial and intestinal mucosae coupled with a rise in circulating antigen-specific IgA and mesangial deposits of IgA and J chain. Coppo et al. [67] have argued that altered mucosal processing of food antigens such as gliadin, a lectin present in gluten, might be involved in the induction of this disease. High serum levels of IgA antigliadin have been reported in IgAN patients [68–70]. Coppo et al. [71] have also demonstrated that mice orally immunized with gliadin or ovalbumin developed glomerular injury with intense glomerular IgA deposition, including antigliadin IgA antibodies. In addition to intrinsic food antigens, food-borne microbial contaminants may also provide an antigenic stimulus in IgAN. Pestka et al. have demonstrated that mice fed meals contaminated with deoxynivalenol developed increased levels of serum IgA, circulating IgA-IC, mesangial IgA deposition, and hematuria, all clinical features of human IgAN [72–75]. However, in human IgAN, there are many studies indicating that the levels of antibodies to food antigens as well as microbial antigens are not any different from normal individuals **[76, 77]**. Therefore, we have to carefully think differences in immune responses to mucosally encountered antigens, such as quality of antibodies or IC between human and mouse.

## 5. Tonsillar B Cells in the Generation of Nephritogenic IgA in IgAN

Considered together, these studies suggest that mucosal encounter with exogenous antigens derived from fungi, bacteria, and viruses could play a key role in the development of IgAN. On the other hand, one can speculate that there is not a single infectious or food antigen which has been uniformly and convincingly associated with the development of IgAN. It remains unclear, however, precisely how all these microbe-related antigens interact with the IgA immune system to trigger disease. It is actually difficult to assess this question using human samples alone. Therefore, we used animal models. We were able to establish a spontaneous IgAN-prone murine model [6, 78] and adopted it for our purpose. We found that human and murine IgAN are regulated, at least partly, by the same genes in addition to the same phenotype of renal damage [78]. Growing evidence from studies of innate immunity may provide a clue. Toll-like receptors (TLRs) are a family of pathogen recognition molecules that discriminate self from nonself (pathogens) and activate suitable defense mechanisms [79]. TLRs on antigen-presenting cells also initiate and modulate adaptive immunity during infection [80]. To assess these questions in reference to IgAN, we examined the pathological roles of TLRs in this IgAN-prone murine model [81]. An association study using an IgAN-prone murine model showed that the progression of murine IgAN was linked to signaling molecules of the TLR myeloid differential protein 88 (MyD88) [81]. We also examined the relationship between the TLR mRNA expression level in splenocytes and disease activity, and found that the severity of glomerular injury in this model was clearly linked only to the degree of MyD88 and TLR9 expression in splenocytes [81]. TLR9 recognizes microbial unmethylated DNA and is expressed mainly by B cells and dendritic cells (DC) in mucosa [82–84]. In fact, intranasal immunization with CpG oligodeoxynucleotide (CpG-ODN), which is known as a ligand for TLR9, aggravated glomerular damage with elevation of the serum IgA level [81], confirming that TLR, and in particular TLR9, is involved in the pathogenesis of murine IgAN.

We have also confirmed that transfer of BM from the IgAN-prone to wildtype control mice results in the development of IgAN [64]. In contrast, BM transfer from wildtype normal mice to the onset-prone mice abrogates glomerular injury and mesangial deposition. These findings suggest that BM may be a reservoir of memory cells capable of synthesizing IgA with a propensity for mesangial deposition and triggering of GN in murine IgAN, as in human IgAN [26, 27]. 

Are these cells responsible for the nephritogenic IgA production reserved only in BM? To approach the possibility that these responsible cells are disseminated to other lymphoid tissues, we transferred whole spleen cells from the IgAN-prone mice into subacute combined immunodeficient (SCID) mice. BMT as well as cell transfer could reconstitute the murine IgAN in SCID mice, suggesting that the responsible cells may be also localized in systemic lymphoid organs (Nakata et al., paper in preparation). Adoptive transfer analyses in the same study showed that CD19^+^ cells are necessary for the development of murine IgAN. CD19 is expressed on follicular DC and B cells. Since CD19^+^ cell transfer induced glomerular IgA deposition with elevation of serum IgA even in SCID mice lacking T and B cells, responsible CD19^+^ cells in this disease may be mainly B cells.

CD19 is present on B cells from earliest recognizable B-lineage cells during development to B cell blast but is lost on maturation to plasma cells [85]. On the other hand, CD 138 is known to be a cell surface marker for plasma cells [86]. Our recent study also showed that CD138^+^ cells are seen in BM of reconstituted IgAN mice whereas those from spleen did not induce glomerular IgA deposition or glomerular lesions in Balb/c mice. Pre-B or immature B cells in murine BM also express CD138; the cells lose their capacity for CD138 expression after emigrating from BM, but re-express it during the final maturation to plasma cells in peripheral lymphoid tissues or spleen. Therefore, the responsible cells may be CD19^+^ B cells but not mature CD 138^+^ plasma cells, at least in murine IgAN. Furthermore, reconstitution of IgAN by cell transfer of spleen cells depleted of the CD90^+^ pan T cell in both murine strains and cell transfer of CD19^+^ cells in SCID mice indicates that the responsible B cells link to the nephritogenic IgA in a T-cell-independent manner. Our recent experimental study using alymphoplasic (aly/aly) mice [87] further supports this notion. Aly/aly mice have no secondary lymphoid tissues or germinal center (GC) [88]. However, BMT from our IgAN-prone mice also reconstituted glomerular IgA deposition and glomerular lesions in aly/aly recipient mice [87]. This finding suggests that somatic hypermutation in GC may not be required for nephritogenic IgA production, at least in murine IgAN. Accordingly, B cells related to the nephritogenic IgA may mature in GC-independent and T-cell-independent manners. Therefore, B-DC interaction should be carefully evaluated. At the very least, both cells express TLR9 and are key players in mucosal immunity.

Does TLR9 contribute to progression of human IgAN? Our previous study demonstrated that two genotypes of *TLR9* have a strong association with the progression of IgAN, indicating the involvement of *TLR9* also in the pathogenesis of human IgAN [81]. We demonstrated that the CC/CT genotype in rs352139 and TT genotype in rs352140 could be risk factors for the progression of IgAN. As mentioned previously, the tonsil can be a major mucosal site in the pathogenesis of human IgAN. Human tonsils contain relatively few IgA-producing cells, which in vitro secrete predominantly monomeric and polymeric IgA1. The contribution of tonsillar IgA1-producing cells to the total pool of IgA produced in the human body must be minimal considering the absolute number of cells in tonsils versus the mucosal tissues such as gut, bone marrow, lymph nodes, and spleen. However, most IgAN patients indeed showed a decrease in serum IgA after tonsillectomy, in association with improvement of clinical manifestations [89–91]. Moreover, tonsillar B cells in IgAN patients produced more GdIgA1 and pIgA than those of chronic tonsillitis or sleep apnea syndrome [52]. Further, tonsillar CD19^+^ B cells in IgAN also showed downregulation of core1*β*GalT expression, which is inversely correlated with estimated glomerular filtration rate [55]. 

Our recent study demonstrates that some IgAN patients exhibited relatively high expression of tonsillar TLR9 mRNA (the TLR9 high group) and showed an earlier, more complete clinical remission than those with low TLR9 expression [92]. Patients whose serum IgA decreased more than the average of enrolled IgAN patients after tonsillectomy (LargeΔIgA) showed higher cumulative remission rates of proteinuria than did the others (SmallΔIgA). CC/CT genotypes in rs352139 [81] were more dominant and tonsillar TLR9 expressions were significantly higher in LargeΔIgA than in SmallΔIgA patients. Therefore, one can speculate that tonsillar B-cell activation via TLR9 may be involved in the production of the nephritogenic GdIgA1 in IgAN. In fact, mononuclear cells from tonsils of IgAN patients treated with CpG-DNA showed increased IgA, IFN*γ*, and the B-cell activating factor of the tumor necrosis factor family (BAFF) production [93]. On the other hand, Kodama et al. reported that B1 cells, which produce IgA in T-cell-independent manner, are increased in tonsils of IgAN patients [94]. Since BAFF enhanced activation of TLR in activated B1 cells [95], an increase of B1 cells may be important for pathogenesis of IgAN. In this regard, it is noteworthy that recent microarray analysis of tonsils from IgAN patients showed that tonsillar expression of APOBEC2, known as a messenger molecule for B cell development, is increased in IgAN [96]. 

Does mucosal DC expressing TLR9 play a role in the pathogenesis of IgAN? To assess whether the TLR9 on mucosal DC and B cells may play different roles in pathogenesis of IgAN, we stimulated the IgAN-prone mice with cell-specific CpG-ODN. Different CpG-ODNs have been used to study cell regulation by TLR9 in DC, and it has been shown that CpG-A-ODN induces large amounts of IFN-*α* in plasmacytoid DC, that CpG-B-ODN acts as a potent stimulant of B cells, and that CpG-C-ODN functions as an activator of both B cells and DC [97–99]. Surprisingly, each CpG-ODN stimulation induced different disease phenotypes in IgAN-prone mice. Serum IgA levels increased in mice treated with CpG-B-ODN, while elevation of serum IgA-IgG2a ICs was found following administration of CpG-A-ODN or CpG-C-ODN. The CpG-A-ODN group showed mesangial proliferation, and CpG-B-ODN therapy induced extracellular matrix expansion. The fluorescent intensity of glomerular IgA and IgG differed in each group. Those data suggest that, at least in murine IgAN, mucosal DC activation via TLR9 may link the IgA/IgG IC formation to subsequent proliferative glomerular damage.

## 6. Conclusion

As demonstrated here, clinical and experimental studies indicate that responsible B cells in the tonsil may link to the production of nephritogenic GdIgA1, which subsequently induces pIgA1 and IgA/IgG IC formation. The B cells seem to be activated by exogenous microbial antigens via TLR9 in mucosa and traffic systemic organs, including BM. Therefore, surgical removal of tonsils may directly decrease the number of the responsible B cells and their activation. Additional steroid pulse therapy may thus further eliminate the responsible B cells that are systemically disseminated.
